# Bioinformatic Exploration of Circulating microRNAs Related to Functional Outcomes in Patients With Acute Ischemic Stroke: An Exploratory Prospective Study

**DOI:** 10.7759/cureus.67476

**Published:** 2024-08-22

**Authors:** Takeshi Imura, Masaru Abiko, Ryo Tanaka

**Affiliations:** 1 Department of Rehabilitation, Hiroshima Cosmopolitan University, Hiroshima, JPN; 2 Department of Neurosurgery, JA Onomichi General Hospital, Onomichi, JPN; 3 Graduate School of Humanities and Social Sciences, Hiroshima University, Higashihiroshima, JPN

**Keywords:** bioinformatics, serum, functional outcome, acute ischemic stroke, microrna

## Abstract

Background

Although epigenetic modifications have been expected to play an important role in neuroplasticity for stroke recovery, the role of dynamic microRNA (miRNA) regulation related to functional outcomes after ischemic stroke remains unclear. Therefore, the current study performed a comprehensive miRNA expression analysis in serum to identify specifically altered circulating miRNAs associated with different grades of functional outcomes in patients with acute ischemic stroke (AIS).

Methods

Twelve patients with AIS in the middle cerebral artery region were included in this study. Peripheral blood samples were collected from patients one or two days after hospitalization. Total RNA, including small RNAs, was extracted from 400 µL of serum, and comprehensive miRNA expression analysis was performed to identify specifically altered circulating miRNAs associated with different grades of functional outcomes. Functional outcomes were evaluated three months after stroke onset using the modified Rankin Scale (mRS), classified as favorable (mRS score of 0 or 1) or unfavorable (mRS score of 2 to 5). Differentially expressed miRNAs were analyzed using the DESeq2 package. Target genes of the miRNAs were explored using miRTargetLink 2.0.

Results

Acute miRNA expression dynamics were characterized by differences in the patients’ functional outcomes following ischemic stroke. The favorable outcome group exhibited significantly downregulated miRNAs, including hsa-miR-218-1, hsa-miR-218-2, hsa-miR-320e, hsa-miR-320d-1, hsa-miR-320d-2, hsa-miR-326, and hsa-miR-4429. In addition, 15 miRNAs, including hsa-miR-223, hsa-miR-18a, hsa-miR-411, and hsa-miR-128-1, were significantly upregulated in the favorable outcome group compared to the unfavorable outcome group. Interesting and strong validated networks between miRNAs and their target genes were identified.

Conclusion

This study identified specifically altered circulating miRNAs in serum associated with varying grades of functional outcomes in AIS patients and explored miRNA-target gene networks that might contribute to these outcomes. Although further studies are needed, this study highlights their potential role as biomarkers for predicting functional outcomes in patients with AIS.

## Introduction

Ischemic strokes account for approximately 70% of all strokes [1]. Occlusion or stenosis of the middle cerebral artery caused by atherothrombotic or cardioembolic events may lead to various residual disabilities. Although the development of acute revascularization treatments such as recombinant tissue plasminogen activator and mechanical thrombectomy has dramatically improved the prognosis of patients with major cerebral artery lesions [2], residual disabilities can often persist. Further advancements in treatment outcomes are essential to enhance patients’ quality of life and minimize social security costs.

Rehabilitation is critical for improving daily functional independence and social participation among patients with confirmed brain infarction lesions and persistent functional impairment or disability, even after revascularization treatment [2]. During the rehabilitation process, the degree of functional recovery can vary considerably between patients who respond to rehabilitative treatment and those who do not [3]. Previous studies have suggested that factors such as the severity of neurological dysfunction, time elapsed from stroke onset, and the volume of the ischemic lesion can affect functional recovery [4]. At the molecular level, Li et al. found that glial growth factor 2 promoted neural functional recovery after ischemic stroke [5]. Although numerous molecules associated with functional recovery after ischemic stroke have been identified [5], the molecular dynamics related to the functional outcomes of ischemic stroke are not yet fully understood.

Epigenetic modifications have been recognized as important gene expression regulators and are expected to play a key role in neuroplasticity for stroke recovery [6]. Among the various epigenetic modifications, microRNAs (miRNAs), which are short non-coding RNAs approximately 23 nucleotides in length, are considered important diagnostic or prognostic biomarkers because they are easily detectable and remain relatively stable in peripheral blood [7]. A previous study compared miRNA expression between acute ischemic stroke (AIS) patients and healthy controls (i.e., those who did not experience a stroke) to examine its role as a diagnostic marker and investigate molecular changes following stroke onset [8]. However, the role of dynamic miRNA regulation related to functional outcomes after ischemic stroke remains unclear due to the lack of studies that compare miRNA expression by different functional outcomes in AIS patients with similar initial stroke severity. Additionally, previous studies have been limited by the fact that they evaluated only selected miRNAs [8], and no study to date has conducted a comprehensive miRNA expression analysis in the context of functional outcomes following ischemic stroke. Therefore, the current study carried out a comprehensive miRNA expression analysis using next-generation sequencing in serum to identify specifically altered circulating miRNAs associated with different grades of functional outcomes in patients with AIS.

## Materials and methods

Patient recruitment and blood sample collection

This study included patients diagnosed with AIS and admitted for treatment to the Department of Neurosurgery, JA Onomichi General Hospital. The inclusion criteria were as follows: (1) ischemic stroke (atherothrombotic or cardioembolic) in the middle cerebral artery region for the first time; (2) Diffusion-Weighted Imaging-Alberta Stroke Program Early Computed Tomography Scores (DWI-ASPECTS) ranging from 5 to 10 (established ischemic lesion after acute revascularization treatment); and (3) age at the time of consent ranging between 20 and 85 years. The exclusion criteria were as follows: (1) Functional Ambulation Categories (FAC) of 3 or less prior to stroke onset or (2) inability to conduct standard neurosurgical treatment or rehabilitation due to serious medical complications during hospitalization. All treatments, including acute revascularization and rehabilitation, were performed in accordance with the Japan Stroke Society Guideline 2021 for the Treatment of Stroke [2]. After acute management at JA Onomichi General Hospital, the patients were transferred to a convalescent rehabilitation hospital as appropriate for continued rehabilitation.

Peripheral blood samples were collected in serum tubes from patients one or two days after hospitalization. The samples were then allowed to clot at room temperature for at least 30 minutes and, after centrifugation at 3500 rpm for 15 minutes at 4°C, were aliquoted into microtubes and frozen at −20°C until required for analysis.

This study was approved by the ethical committees of Hiroshima Cosmopolitan University (approval number: 2020008) and JA Onomichi General Hospital (approval number: OJH-202192). Written informed consent was obtained from all patients, and the study was conducted in accordance with the Declaration of Helsinki.

Ischemic lesion evaluation and functional assessment

Evaluation of established ischemic lesions was carried out by an expert vascular neurosurgeon (M.A.) blinded to miRNA expression, using the DWI-ASPECTS. Patients who underwent acute revascularization therapy to minimize ischemic lesions were evaluated with the score after completion of treatment. Imaging data was acquired using a 3-T MRI scanner (GE Healthcare Japan).

Functional stroke severity upon acute rehabilitation admission was evaluated by a nurse blinded to the patient’s DWI-ASPECTS score and miRNA expression, using the National Institutes of Health Stroke Scale (NIHSS). Functional outcome evaluation was conducted by an expert vascular neurosurgeon (M.A.) blinded to miRNA expression during the patient’s follow-up appointment at JA Onomichi General Hospital three months after stroke onset, using the modified Rankin Scale (mRS). The mRS at three months has generally been used as the functional outcome following AIS [9,10]. The functional outcomes were classified as favorable (mRS score of 0 or 1) or unfavorable (mRS score of 2 to 5), in accordance with previous studies [9,10].

RNA extraction and quality check

The MicroRNA Isolation Kit (BioChain) was used to extract and purify total RNA, including small RNAs, from 400 µL of serum, in accordance with the manufacturer’s protocol. The quality and quantity of the extracted small RNAs were assessed using NanoDrop (Thermo Fisher Scientific, Waltham, Massachusetts), the QuantiFluor RNA System (Promega, Madison, Wisconsin), and the Agilent Small RNA kit of the BioAnalyzer 2100 System (Agilent Technologies, Santa Clara, California), as per the manufacturer’s instructions. RNA integrity number ≥ 7 and A260/A280 and A260/230 ratios ≥ 1.8 were set as the quality check criteria. Based on miRNA concentration, 10 of the 12 samples were selected for preparation of a small RNA library for next-generation sequencing analysis, and the remaining 2 samples were excluded from further analysis.

Preparation of a small RNA library and comprehensive miRNA sequencing analysis

Small RNA library construction and sequencing analysis were performed according to our previous study [7]. We prepared the miRNA sequencing library using a QIAseq miRNA Library Kit (Qiagen, Hilden, Germany) in accordance with the manufacturer’s protocols. At least 1 ng of total RNA was used for library construction. A pre-adenylated DNA adapter or an RNA adapter was ligated to the 3’ ends of all miRNAs or to the 5’ ends of mature miRNAs, respectively. For reverse transcription (RT), an RT primer with Unique Molecular Indices was used. Finally, sample-specific indexed primers were used for amplification of the library, and 75 nt single reads were generated through sequencing on a NextSeq 500 sequencer (Illumina, San Diego, California). Seqkit (version 0.10.1, Shanghai, China) was used to remove duplicated reads from the raw reads. Next, cutadapt (version 3.7, Marcel Martin, Tübingen, Germany) was used to trim the adapter sequence. The trimmed reads were mapped to the human gencode.v38 and the human GRCh38 genome using STAR (version 2.7.9a) with the following parameters: "--alignEndsType EndToEnd", "--outFilterMismatchNmax 1", "--outFilterMultimapScoreRange 0", "--outReadsUnmapped None", "--outSAMtype BAM Unsorted", "--outFilterMultimapNmax 10", "--outSAMunmapped Within", "--outFilterScoreMinOverLread 0", "--outFilterMatchNminOverLread 0", "--outFilterMatchNmin 16", "--alignSJDBoverhangMin 1000", and "--alignIntronMax 1". FeatureCounts in the Subread package (version 2.0.1) with the parameters: -s 1, -O, -M, -p, -C, -B, and -T was used for quantification of the reads that mapped to miRNAs. The miRBase (https://www.mirbase.org/) was used as the database.

Identification of differentially expressed miRNAs and exploration of their target genes

Differentially expressed miRNAs (DEMs) were analyzed using the DESeq2 package (version 1.20.0) to identify miRNAs that were uniquely altered in the favorable outcome group compared to the unfavorable outcome group. The thresholds for significant DEMs were set at |log2FC| >1.5 and P value <0.05. Significant DEMs with log2FC >1.5 or log2FC <−1.5 were categorized as upregulated or downregulated DEMs, respectively, in accordance with a previous study [7]. We used Shinyheatmap (http://shinyheatmap.com/) to display the heatmap of DEMs. Target genes of the miRNAs that have been reported to be associated with functional recovery after stroke in previous studies were explored using miRTargetLink 2.0 (https://ccb-compute.cs.uni-saarland.de/mirtargetlink2). We selected "strong validated only" when presenting the interaction between miRNAs and target genes using miRTargetLink 2.0.

Functional enrichment analysis

Gene Ontology (GO) and Kyoto Encyclopedia of Genes and Genomes (KEGG) pathway enrichment analyses were performed using the Database for Annotation, Visualization, and Integrated Discovery (DAVID) (https://david.ncifcrf.gov/) to elucidate the biological implications and functions of the significantly downregulated or upregulated DEMs. Statistical significance was set at P value < 0.05. We applied the Benjamini-Hochberg procedure to adjust P values. GO enrichment analysis included three domains: biological process, cellular component, and molecular function. We used Metascape (https://metascape.org/) to display the bar graph of enriched terms.

Statistical analysis

All statistical analyses were performed using JSTAT (Sato, Japan) and R (version 3.6.1, R Foundation, Vienna, Austria) software. The Mann-Whitney U test was used for the comparison of age, DWI-ASPECTS, and NIHSS upon rehabilitation admission, while Fisher’s exact test was used to compare sex, ischemic stroke type, and acute revascularization treatment between groups. A P-value <0.05 was considered statistically significant.

## Results

Patient demographics

Of the 12 serum samples collected from patients with AIS, 2 were excluded from the sequencing analysis due to low miRNA concentrations. Of the 10 patients, 4 were classified as the unfavorable outcome group (2 patients with mRS 3 and 2 patients with mRS 2) and 6 were classified as the favorable outcome group (3 patients with mRS 0 and 3 patients with mRS 1) according to the mRS at 3 months after stroke onset. The mean age of the patients did not significantly differ between the groups (unfavorable outcome group: 77.75±7.93 years; favorable outcome group: 76.83±4.83 years; Table 1). All patients in the unfavorable outcome group were female, while 4 out of 6 patients in the favorable outcome group were female. There were no significant differences in ischemic stroke type between the groups (unfavorable outcome group: 2 patients with atherothrombotic and 2 with cardioembolic; favorable outcome group: 1 patient with atherothrombotic and 5 with cardioembolic) or in the acute revascularization treatment. Established ischemic lesions (DWI-ASPECTS scores; unfavorable outcome group: 7.25±1.71; favorable outcome group: 8.33±1.86) and functional stroke severity upon acute rehabilitation admission (NIHSS scores; unfavorable outcome group: 9.50±2.38; favorable outcome group: 8.67±6.62) also did not differ significantly between the groups.

**Table 1:** Baseline characteristics of subjects DWI-ASPECTS, Diffusion-Weighted Imaging-Alberta Stroke Program Early Computed Tomography Score; DWI, diffusion-weighted imaging; MT, mechanical thrombectomy; NIHSS, National Institutes of Health Stroke Scale; SD, standard deviation; t-PA, tissue plasminogen activator. *The Mann-Whitney U test was applied. **The Fisher’s Exact test was applied.

Demographics	Unfavorable outcome group	Favorable outcome group	Significance
Age, years (mean ± SD)	77.75±7.93	76.83±4.83	P = 0.48^*^
Sex, n (%)			P = 0.47^**^
Male	0 (0%)	2 (33.3%)	
Female	4 (100%)	4 (66.7%)	
Ischemic stroke type, n (%)			P = 0.50^**^
Atherothrombotic	2 (50%)	1 (16.7%)	
Cardioembolic	2 (50%)	5 (83.3%)	
Acute revascularization treatment, n (%)			P = 0.71^**^
MT	1 (25%)	3 (50%)	
t-PA and MT	0 (0%)	1 (16.7%)	
Not performed	3 (75%)	2 (33.3%)	
DWI-ASPECTS	7.25±1.71	8.33±1.86	P = 0.48^*^
NIHSS upon rehabilitation admission	9.50±2.38	8.67±6.62	P = 0.48^*^

Identification of differentially expressed miRNAs and experimentally validated target genes

**Figure 1:**
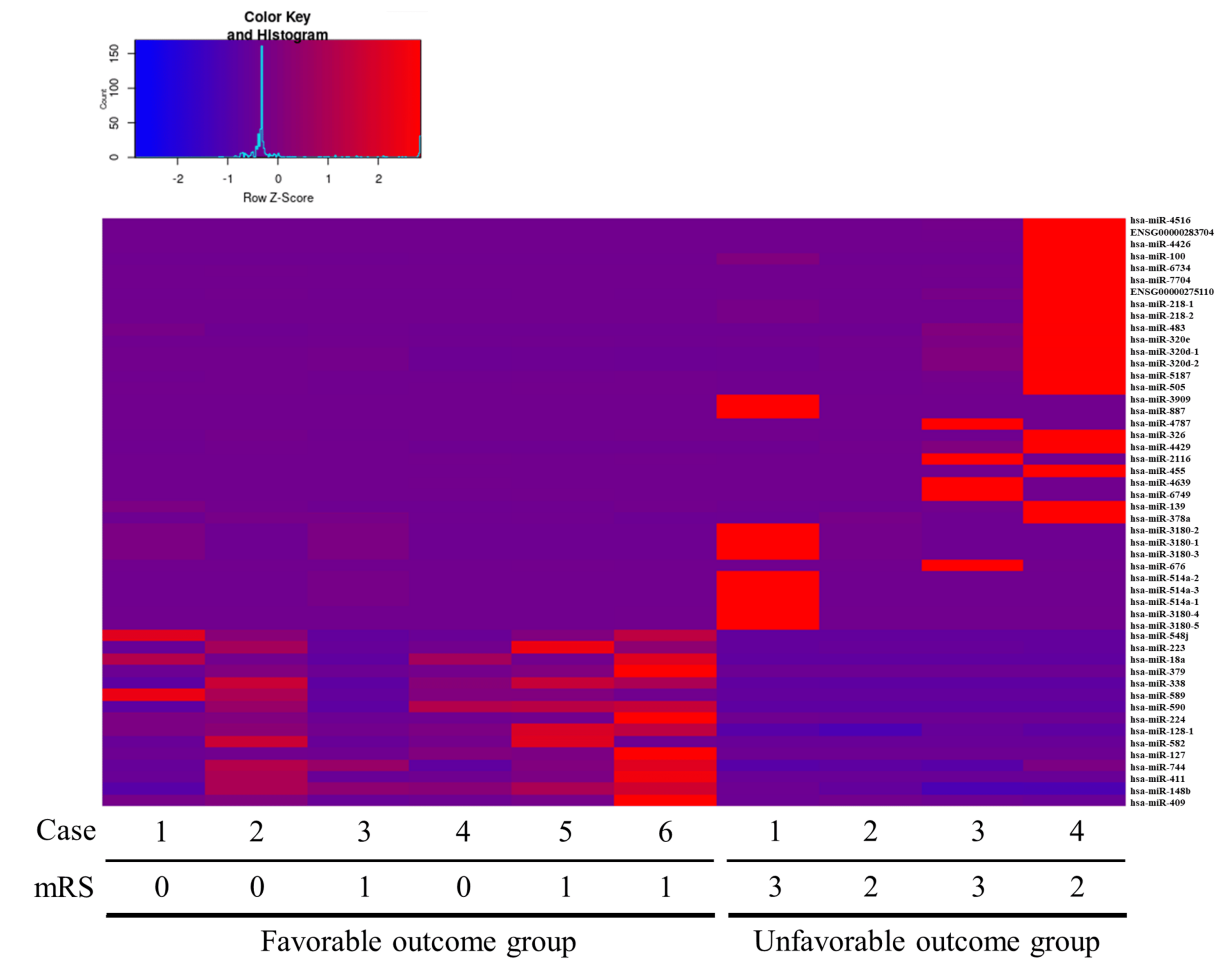
The heatmap of differentially expressed miRNAs between favorable outcome group and unfavorable outcome group 35 miRNAs from hsa-miR-4516 to hsa-miR-3180-5 were identified as downregulated miRNAs and 15 miRNAs from hsa-miR-548j to hsa-miR-409 were identified as upregulated miRNAs in favorable outcome group.

**Figure 2:**
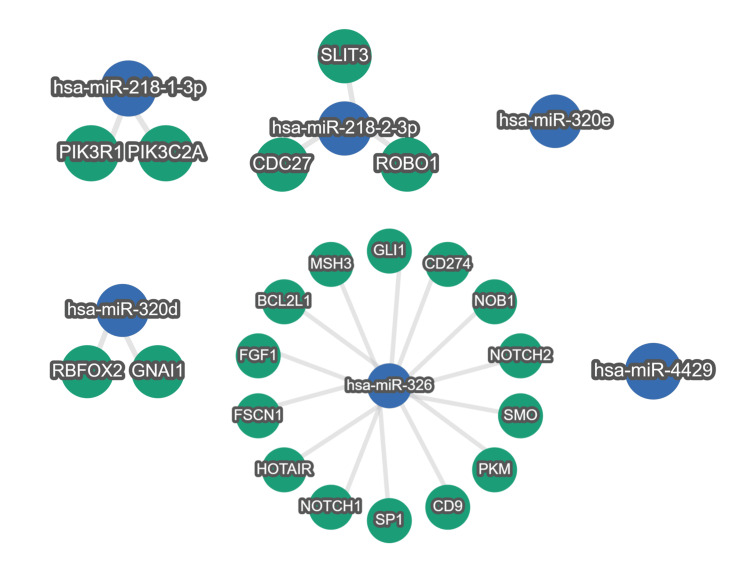
Downregulated miRNA-target gene network Interactions between downregulated miRNAs and strong validated target genes are displayed.

**Figure 3:**
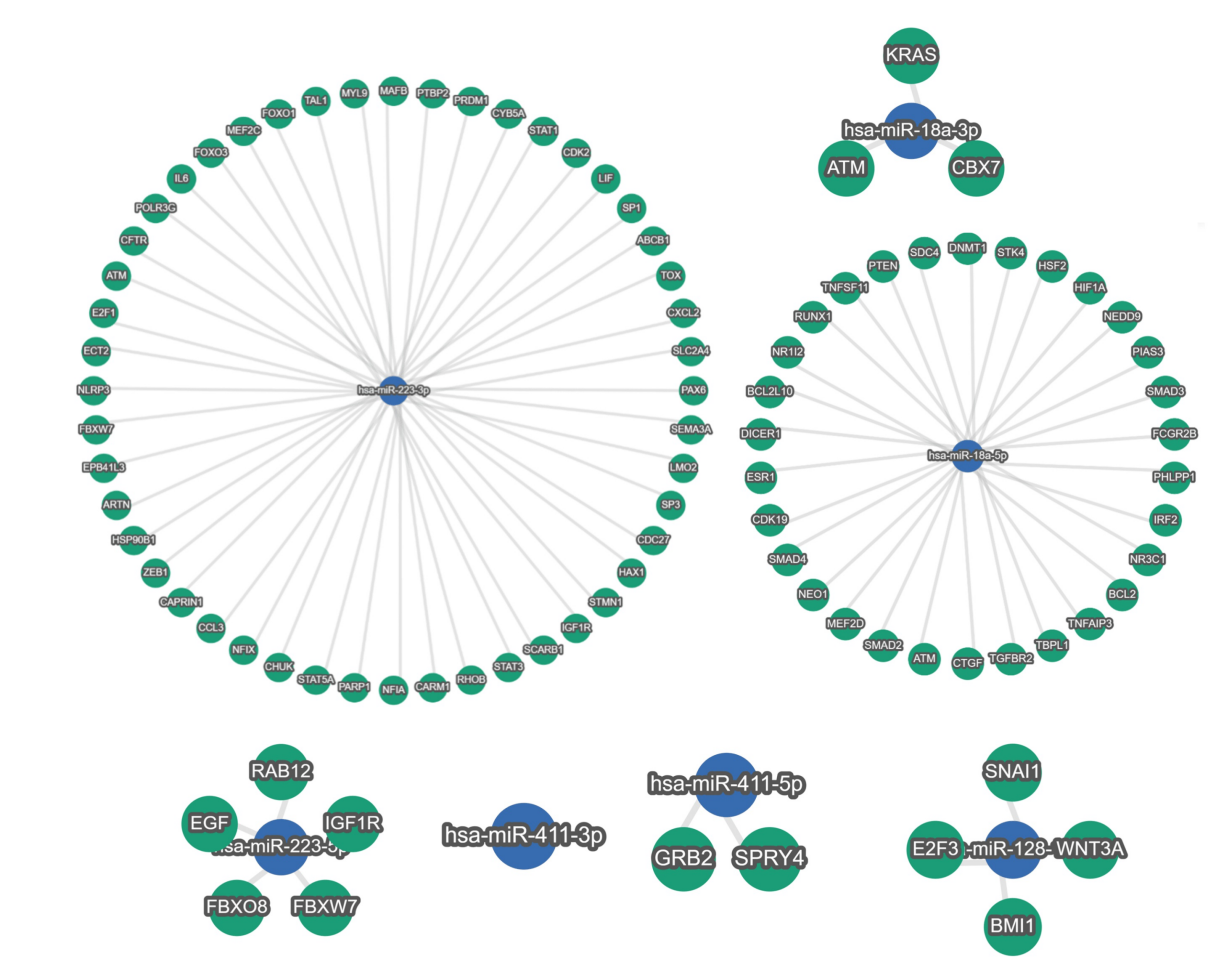
Upregulated miRNA-target gene network Interactions between upregulated miRNAs and strong validated target genes are displayed.

Sequencing analysis confirmed the expression of 997 miRNAs out of a total of 1,879 miRNAs registered in the miRBase. The miRNA expression profiles differed significantly between the unfavorable and favorable outcome groups. Comparison of miRNA expression between groups showed that 35 miRNAs, including hsa-miR-218-1, hsa-miR-218-2, hsa-miR-320e, hsa-miR-320d-1, hsa-miR-320d-2, hsa-miR-326, and hsa-miR-4429, were significantly downregulated in the favorable outcome group compared to the unfavorable outcome group (Figure 1 and Table 2). Additionally, 15 miRNAs, including hsa-miR-223, hsa-miR-18a, hsa-miR-411, and hsa-miR-128-1, were significantly upregulated in the favorable outcome group compared to the unfavorable outcome group (Figure 1 and Table 3). Target genes of downregulated or upregulated DEMs that have been reported to be associated with functional recovery after stroke in previous studies were explored. The strongly validated networks between miRNAs and their target genes are represented in Figures 2, 3.

**Table 2:** Significantly downregulated miRNAs in the favorable outcome group compared to the unfavorable outcome group The Wald test was applied. FC, fold change.

MicroRNA	Average read count (unfavorable outcome group)	Average read count (favorable outcome group)	Log2FC	P-value
hsa-miR-4516	5510.6	6.2	-10.86	<0.0001
ENSG00000283704	5571.6	14	-8.98	<0.0001
hsa-miR-4426	6109.3	0.2	-12.03	<0.0001
hsa-miR-100	4786.8	12.3	-8.37	<0.0001
hsa-miR-6734	21063.6	130.1	-7.84	0.0001
hsa-miR-7704	3727.7	4	-9.05	0.0002
ENSG00000275110	18844	283.9	-6.55	0.0003
hsa-miR-218-1	1942.3	9.3	-7.27	0.0003
hsa-miR-218-2	1942.3	9.3	-7.27	0.0003
hsa-miR-483	114968	5135.8	-5.09	0.0023
hsa-miR-320e	3135.8	73.1	-4.37	0.0032
hsa-miR-320d-1	28660.9	2749.4	-3.79	0.0050
hsa-miR-320d-2	27904.7	2726.3	-3.72	0.0056
hsa-miR-5187	11249.9	285	-5.69	0.0059
hsa-miR-505	17335.5	639.3	-5.26	0.0068
hsa-miR-3909	127	0	-7.82	0.0110
hsa-miR-887	163.5	0	-7.65	0.0128
hsa-miR-4787	160.9	0	-7.54	0.0142
hsa-miR-326	13876	564.9	-5.11	0.0156
hsa-miR-4429	2197.7	92.1	-4.01	0.0160
hsa-miR-2116	265.4	0.9	-7.07	0.0210
hsa-miR-455	715.9	0.2	-6.63	0.0257
hsa-miR-4639	167.9	0.7	-6.68	0.0296
hsa-miR-6749	135.2	0.7	-6.51	0.0341
hsa-miR-139	22026	2742.4	-3.67	0.0366
hsa-miR-378a	7424.5	1222.1	-2.57	0.0391
hsa-miR-3180-2	34.9	4.1	-6.29	0.0414
hsa-miR-3180-1	32.6	3.8	-6.29	0.0414
hsa-miR-3180-3	32.6	3.8	-6.29	0.0414
hsa-miR-676	153.7	1.2	-6.24	0.0416
hsa-miR-514a-2	49.2	2.2	-6.21	0.0434
hsa-miR-514a-3	49.2	2.2	-6.21	0.0434
hsa-miR-514a-1	44.2	2	-6.21	0.0434
hsa-miR-3180-4	18.7	0.1	-6.21	0.0441
hsa-miR-3180-5	18.7	0.1	-6.21	0.0441

**Table 3:** Significantly upregulated miRNAs in the favorable outcome group compared to the unfavorable outcome group The Wald test was applied. FC, fold change.

MicroRNA	Average read count (unfavorable outcome group)	Average read count (favorable outcome group)	Log2FC	P-value
hsa-miR-548j	0	972	5.99	0.0006
hsa-miR-223	105486.1	983121.5	3.33	0.0010
hsa-miR-18a	0	1044.3	5.55	0.0018
hsa-miR-379	0	1634	5.96	0.0052
hsa-miR-338	4.1	1335.1	5.29	0.0052
hsa-miR-589	5.6	742.4	4.53	0.0053
hsa-miR-590	0	749.1	5.49	0.0079
hsa-miR-224	179.8	8462.3	4.84	0.0102
hsa-miR-128-1	1306.2	6876.7	2.55	0.0232
hsa-miR-582	0	571.4	4.97	0.0245
hsa-miR-127	0	420.1	4.61	0.0269
hsa-miR-744	1383.2	4129.6	2.01	0.0295
hsa-miR-411	0	493.6	4.62	0.0383
hsa-miR-148b	5013.8	16937	1.74	0.0386
hsa-miR-409	880.9	7199.7	2.70	0.0470

Functional enrichment analysis

**Figure 4:**
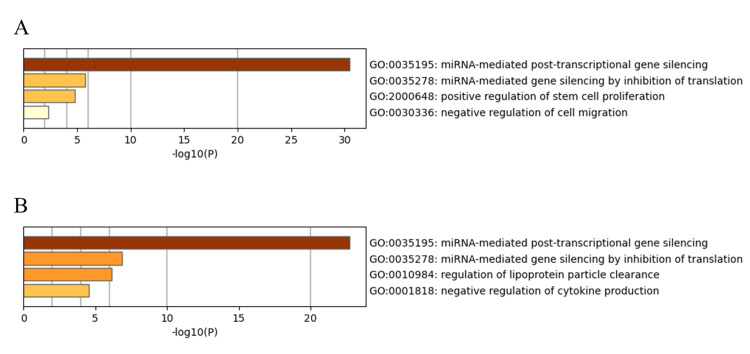
Bar graph of enriched terms via the Metascape Bar graph of enriched terms of 35 downregulated miRNAs (A) and 15 upregulated miRNAs (B).

Figure 4A shows the bar graph of enriched terms for 35 downregulated miRNAs, and Figure 4B shows those for 15 upregulated miRNAs via Metascape. Enrichment analysis was performed by annotating the categories "GOTERM" and "KEGG_PATHWAY" via DAVID to understand the biological implications of the 35 downregulated (Table 4) and 15 upregulated DEMs (Table 5). Both downregulated and upregulated DEMs were commonly enriched in "gene silencing by miRNA," "miRNA-mediated inhibition of translation," "mRNA cleavage involved in gene silencing by miRNA," "RISC complex," "mRNA binding involved in posttranscriptional gene silencing," and "mRNA 3'-UTR binding" in the GOTERM category and "MicroRNAs in cancer" in the KEGG_PATHWAY category. In the GOTERM category, downregulated DEMs were specifically enriched in "positive regulation of stem cell proliferation," "long-term synaptic potentiation," "cellular response to glucose stimulus," and "extracellular vesicle," while upregulated DEMs were specifically enriched in "negative regulation of epithelial to mesenchymal transition" and "extracellular space."

**Table 4:** Enrichment analysis for the downregulated miRNAs in the favorable outcome group compared to the unfavorable outcome group BP, biological process; CC, cellular component; GO, Gene Ontology; KEGG, Kyoto Encyclopedia of Genes and Genomes; MF, molecular function.

Category	Term	Count (%)	P-value	Fold enrichment
GOTERM_BP_DIRECT	gene silencing by miRNA	21 (63.6%)	<0.0001	33.2
GOTERM_BP_DIRECT	miRNA-mediated inhibition of translation	4 (12.1%)	0.0027	37.05
GOTERM_BP_DIRECT	positive regulation of stem cell proliferation	3 (9.1%)	0.0112	65.5
GOTERM_BP_DIRECT	long-term synaptic potentiation	3 (9.1%)	0.0206	41.68
GOTERM_BP_DIRECT	cellular response to glucose stimulus	3 (9.1%)	0.0235	34.82
GOTERM_BP_DIRECT	mRNA cleavage involved in gene silencing by miRNA	3 (9.1%)	0.031	27.51
GOTERM_CC_DIRECT	RISC complex	12 (36.4%)	<0.0001	39.75
GOTERM_CC_DIRECT	extracellular vesicle	4 (12.1%)	0.0003	34.63
GOTERM_MF_DIRECT	mRNA binding involved in posttranscriptional gene silencing	11 (33.3%)	<0.0001	63.79
GOTERM_MF_DIRECT	mRNA 3'-UTR binding	5 (15.2%)	<0.0001	34.61
KEGG_PATHWAY	MicroRNAs in cancer	3 (9.1%)	0.0013	27.94

**Table 5:** Enrichment analysis for the upregulated miRNAs in the favorable outcome group compared to the unfavorable outcome group BP, biological process; CC, cellular component; GO, Gene Ontology; KEGG, Kyoto Encyclopedia of Genes and Genomes; MF, molecular function.

Category	Term	Count (%)	P-value	Fold enrichment
GOTERM_BP_DIRECT	gene silencing by miRNA	13 (86.7%)	<0.0001	30.83
GOTERM_BP_DIRECT	miRNA-mediated inhibition of translation	4 (26.7%)	0.0012	55.57
GOTERM_BP_DIRECT	negative regulation of epithelial-to-mesenchymal transition	3 (20.0%)	0.0093	89.7
GOTERM_BP_DIRECT	mRNA cleavage involved in gene silencing by miRNA	3 (20.0%)	0.0326	41.26
GOTERM_CC_DIRECT	RISC complex	13 (86.7%)	<0.0001	49.69
GOTERM_CC_DIRECT	extracellular space	11 (73.3%)	<0.0001	8.99
GOTERM_MF_DIRECT	mRNA binding involved in posttranscriptional gene silencing	12 (80.0%)	<0.0001	63.79
GOTERM_MF_DIRECT	mRNA 3'-UTR binding	7 (46.7%)	<0.0001	44.42
KEGG_PATHWAY	MicroRNAs in cancer	4 (26.7%)	<0.0001	27.94

## Discussion

The current study carried out a comprehensive miRNA expression analysis using miRNA sequencing to explore specifically altered circulating miRNAs by functional outcomes in patients with AIS. Additionally, miRNA-target gene networks that might be associated with functional outcomes were explored. The present findings suggest that acute miRNA expression dynamics are characterized by differences in the patient’s functional outcomes following an ischemic stroke.

The favorable outcome group exhibited 35 miRNAs, including hsa-miR-218-1, hsa-miR-218-2, hsa-miR-320e, hsa-miR-320d-1, hsa-miR-320d-2, hsa-miR-326, and hsa-miR-4429, that were significantly downregulated, and these DEMs were enriched in “positive regulation of stem cell proliferation,” “long-term synaptic potentiation,” “cellular response to glucose stimulus,” and “extracellular vesicle.” Among the downregulated miRNAs confirmed to be associated with functional recovery from stroke based on the findings of previous studies, hsa-miR-218-2 and hsa-miR-326 had target genes related to functional recovery. A positive correlation between the plasma miR-218 levels of patients with AIS and their NIHSS scores has been reported previously [8]. Interestingly, target gene analysis using miRTargetLink 2.0 revealed the hsa-miR-218-2-3p-ROBO1 network and the hsa-miR-218-2-3p-SLIT3 network. Previous studies have reported the roles of ROBO1 and SLIT3 in axon guidance of neurons [11,12], which might influence the functional outcomes observed in the present study.

Liang et al. investigated the role of miR-320 in cerebral ischemia/reperfusion (I/R) injury using a mouse model and oxygen-glucose deprivation (OGD)-treated PC12 cells [13]. Interestingly, miR-320 was found to enhance cell apoptosis in the cortical infarcted peripheral zone and increase brain infarction or edema volume in the mouse model, while miR-320 inhibitors decreased the risk of these negative events [13]. The miRTargetLink 2.0 analysis showed that hsa-miR-326 had networks with FGF1, SP1, and GLI1. FGF1 and SP1 are known as effective neuroprotective factors, including their role in reducing brain edema [14,15]. Additionally, Xiao et al. reported that GLI1 is related to axon guidance and neurogenesis in acute ischemic stroke model mice [16]. Although the role of miR-326 in ischemic brain injury remains unclear, this study identified the hsa-miR-326-FGF1 network, hsa-miR-326-SP1 network, and hsa-miR-326-GLI1 network, which may be associated with functional outcomes after ischemic stroke. The current study identified specifically downregulated miRNAs with dynamics that are consistent with the biological roles reported in previous studies, suggesting that they could be involved in functional recovery after ischemic brain pathogenesis and, consequently, contribute to better functional outcomes.

The favorable outcome group also exhibited 15 miRNAs, including hsa-miR-223, hsa-miR-18a, hsa-miR-411, and hsa-miR-128-1, that were significantly upregulated, and these DEMs were significantly enriched in “negative regulation of epithelial to mesenchymal transition” and “extracellular space.” Interestingly, all the upregulated miRNAs confirmed to be associated with functional recovery from stroke based on the findings of previous studies [17-20] had target genes related to functional recovery. Harraz et al. found that overexpression of miR-223 lowered GluR2 and NR2B levels, inhibited NMDA-induced calcium influx in hippocampal neurons, and protected the brain from neuronal cell death after transient global ischemia and excitotoxic injury [17]. The miRTargetLink 2.0 analysis showed that hsa-miR-223-3p had networks with FOXO1, NLRP3, HSP90B1, STAT3, PARP1, RHOB, and SEMA3A. Inhibition of FOXO1, NLRP3, HSP90, or STAT3 has been reported to promote functional recovery after brain injury [21-24]. Additionally, inhibition of PARP1 has neuroprotective effects, such as reducing infarct volume and brain swelling [25], and RHOB impacts neuronal death in transient ischemic model mice [26]. Moreover, Hira et al. reported that inhibition of SEMA3A promotes axonal elongation [27]. Zhang et al. demonstrated that miR-18a is one of the more enriched miRNAs, compared to other members of the miR-17-92 cluster, in the distal axons of sympathetic neurons, implying that it may regulate genes associated with axonal outgrowth [18]. Regarding the hsa-miR-18a-target gene network, the hsa-miR-18a-3p-CBX7 network and hsa-18a-5p-PTEN network were identified. Interestingly, inhibition of PTEN or CBX7 has been related to the improvement of motor function or cognitive function in cerebral ischemic model rats, respectively [28,29].

Gong et al. conducted a study investigating the role of miR-411 using OGD-treated PC12 cells and a spinal cord injury rat model [19]. They found that a miR-411 mimic could dramatically reduce the increased percentage of apoptotic cells caused by OGD and significantly elevate neurological function in the spinal cord injury model rat. The miRTargetLink 2.0 analysis showed that hsa-miR-411-5p had a network with SPRY4, and siRNA-mediated downregulation of SPRY2/4 diminished ischemic brain injury [30]. Karam et al. highlighted the possibility of miR-128 promoting the maturation of neural precursor cells that were already committed to the neural fate [20]. It has also been suggested that the miR-128/nonsense-mediated mRNA decay circuit could directly impact synaptic plasticity, thus playing a significant role in processes such as cognition and memory [20]. Regarding the hsa-miR-128-1-5p-target gene network, the hsa-miR-128-1-5p-E2F3 network and hsa-miR-128-1-5p-WNT3A network were identified. It has been reported that downregulation of E2F3 decreased neuronal cell death [31]. Additionally, Ma et al. suggested that the downregulation of WNT3A was associated with functional recovery in ischemic brain injury model rats [32]. The specific upregulated miRNA dynamics observed in the favorable outcome group could potentially reflect the biological processes related to functional outcomes following ischemic stroke, suggesting their potential role as biomarkers for the prediction of functional outcomes in patients.

This study had several limitations. Firstly, it was conducted as an exploratory investigation with a small sample and aimed to identify specifically altered miRNAs (out of an enormous number of miRNAs) related to functional outcomes in AIS patients using next-generation sequencing analysis. Hence, validation analysis using real-time PCR was not performed. A unique feature of this study is the recruitment of patients with relatively similar infarction sizes among those with middle cerebral artery infarction to examine differences in prognosis, which contributed to the small sample size. Secondly, functional analysis of DEMs in vitro was not performed in the present study, and the biological interpretation of downregulated or upregulated DEMs was based on the findings of previous studies. As a result, there were several DEMs whose biological roles related to functional outcomes following ischemic stroke could not be interpreted, necessitating future studies to investigate their relationship with functional outcomes after ischemic stroke. Thirdly, although no significant differences in baseline characteristics were observed between the groups, we could not rule out the possibility that many other confounding factors such as previous health history, acute revascularization treatment, rehabilitation treatment, etiology of stroke, differences in the timing of sample collection, and actual lesion volume affected the basal expression of miRNAs or functional outcomes. Additionally, the small sample size may have prevented the identification of the effects of heterogeneity between the groups on the results. Fourth, we could not discuss the relationship between the level of microRNAs and post-stroke recovery due to the collection of samples at only one time point. Fifth, we did not account for isoform differences in the comprehensive miRNA analysis, which may hinder comparisons in future studies. Due to these limitations, the results of this study should be interpreted with caution and do not allow for a definitive conclusion. Despite these limitations, and to the best of our knowledge, this is the first study to use comprehensive miRNA expression analysis to explore specifically altered circulating miRNAs associated with varying grades of functional outcomes in patients with AIS of similar initial stroke severity. Further studies investigating their application as therapeutic targets or prognostic biomarkers in AIS patients are necessary.

## Conclusions

This study identified specifically altered circulating miRNAs in serum associated with varying grades of functional outcomes in AIS patients using comprehensive miRNA sequencing analysis and explored miRNA-target gene networks. The patients with favorable outcomes exhibited downregulation of 35 miRNAs, including hsa-miR-218-1, hsa-miR-218-2, hsa-miR-320e, hsa-miR-320d-1, hsa-miR-320d-2, hsa-miR-326, and hsa-miR-4429, and upregulation of 15 miRNAs, including hsa-miR-223, hsa-miR-18a, hsa-miR-411, and hsa-miR-128-1. Although further studies are needed, the findings of this study suggest potential roles of miRNAs as therapeutic targets and biomarkers for the prediction of functional outcomes in patients with AIS.
